# Hypoglycemic mechanism of intestinal bypass surgery in type 2 diabetic rats

**DOI:** 10.1038/s41598-021-98714-1

**Published:** 2021-11-03

**Authors:** Siqi Xie, MingChang Wang, Bin Zhang, ShanGeng Weng

**Affiliations:** 1 Hepatopancreatobiliary Surgery Department, The First Affiliated Hospital of Fujian Medical University, National Abdominal Surgery Institute of Fujian, No 20 Chazhong Road, Fuzhou City, Fujian People’s Republic of China; 2 Department of Intensive Care Unit, The Second Affiliated Hospital of Fujian Medical University, No. 34, North Zhongshan Road, Licheng District, Quanzhou City, Fujian People’s Republic of China

**Keywords:** Type 2 diabetes, Gastrointestinal models

## Abstract

To investigate the effect of duodenal-jejunal bypass (DJB) surgery on postoperative blood glucose in type 2 diabetic rats, and further explore possible mechanisms for the effect of surgical treatment of type 2 diabetes. Forty rats with type 2 diabetes were randomly assigned to 4 groups (n = 10 rats per group), which subsequently underwent DJB, new biliopancreatic diversion (NBPD) or duodenal-jejunal exclusion (DJE) surgery or a sham operation (SHAM). Fasting glucose, 2-h postprandial glucose and blood lipids were measured, and the mRNA in liver and intestinal tissue for bile acid receptor (FXR), as well as the FXR protein expression in the liver tissues were determined. Postprandial blood glucose and fasting TG and FFA in the DJB and NBPD groups were significantly lower than those in the SHAM group and preoperative (*p* < 0.05) at 8 weeks postoperation. Liver FXR protein was expressed at significantly higher in the DJB and NBPD groups than in the other two (*p* < 0.05), and the intestinal FXR mRNA in the DJE group were highest. DJB up-regulates the expression of bile acid receptors in the liver and down-regulates those receptors in the intestinal tract via biliopancreatic diversion. This process reduces TG levels, and subsequently any lipotoxicity to islet cells to produce a hypoglycemic effect.

## Introduction

**Figure 1:**
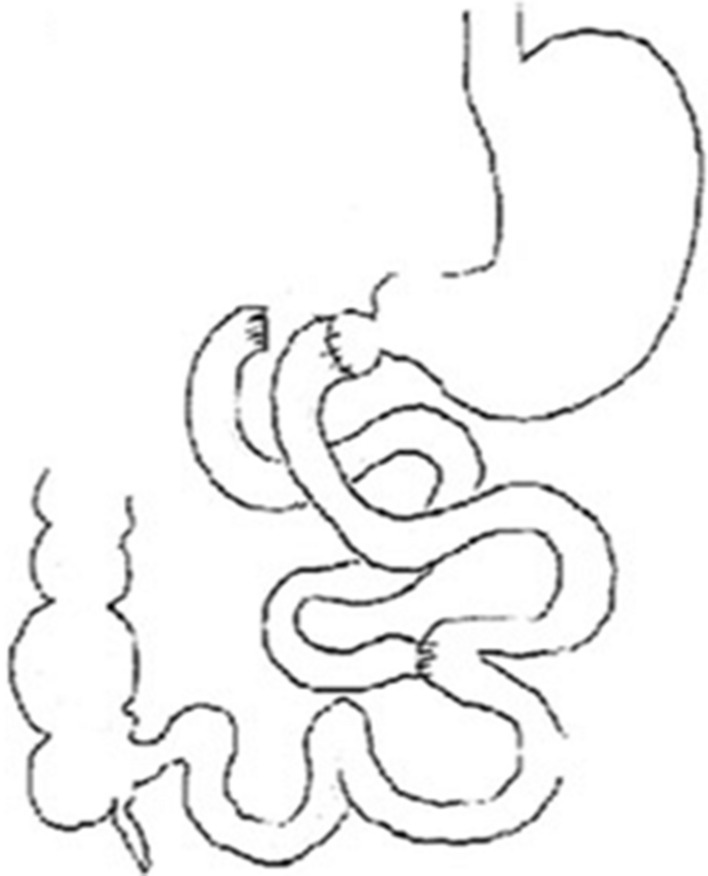
Duodenal-jejunal bypass, DJB.

**Figure 2:**
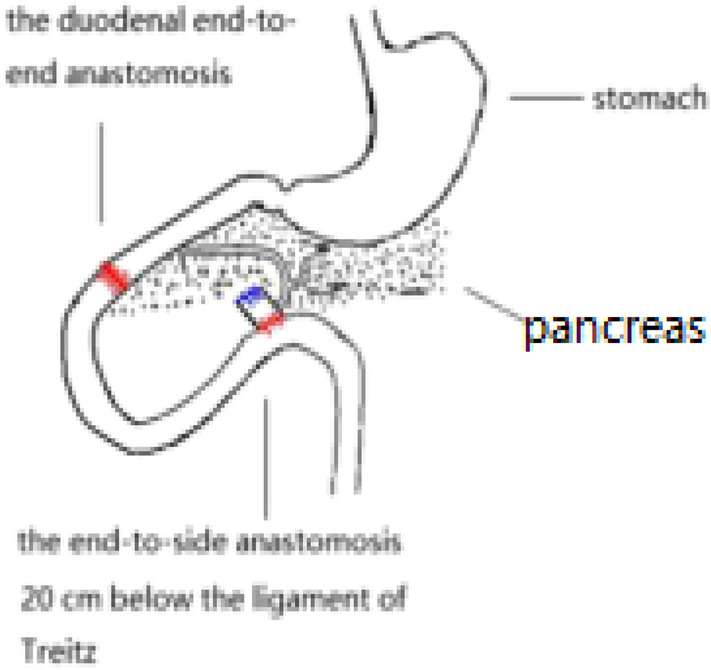
New biliopancreatic diversion, NBPD.

**Figure 3:**
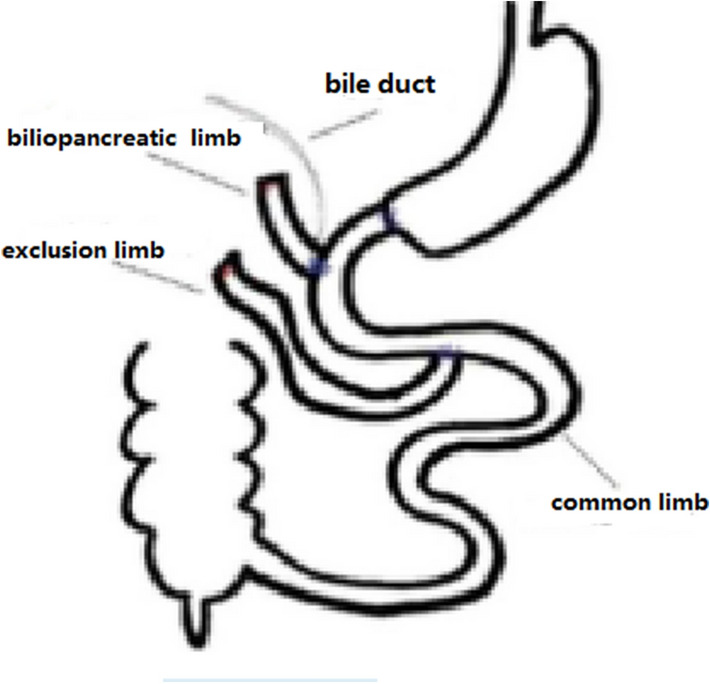
Duodenal-jejunal exclusion, DJE.

**Figure 4:**
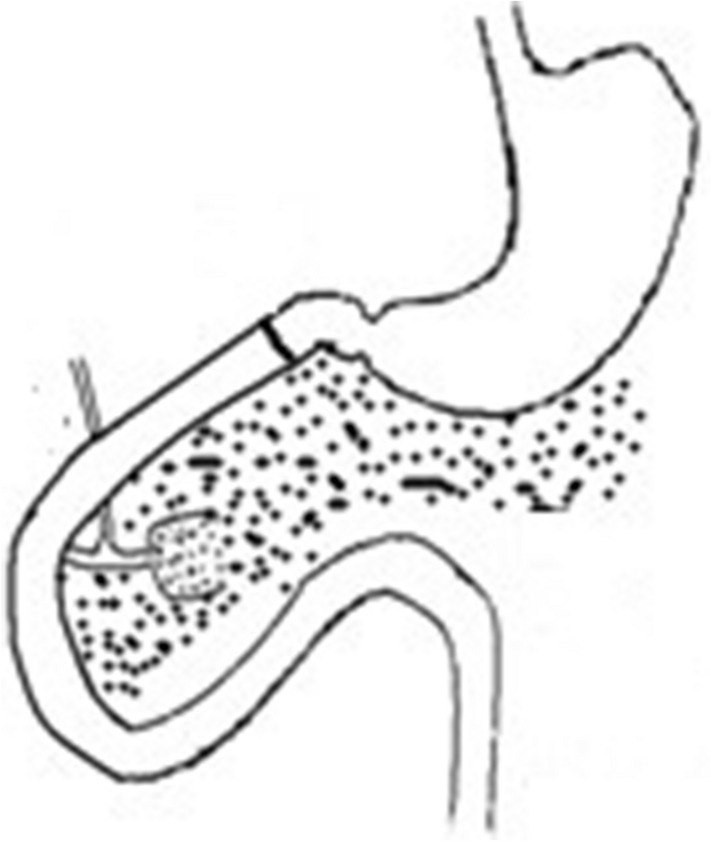
Immediately re-anastomosed after transection, Sham.

The number of people with type 2 diabetes mellitus (T2DM) has increased over the years, and medical treatment has been the main therapeutic approach for T2DM. The significant hypoglycemic effect of surgical weight-loss procedures has been proven in recent years, and these procedures were officially included in the 2009 American Diabetes Association (ADA) guidelines for obese patients with T2DM, with their indications being extended in 2016^1^. The positive effect of surgical treatment on diabetes has been confirmed by years of studies; however, the specific mechanism for its effect remains unclear. Duodenal-Jejunal Bypass (DJB, Fig. 1), a modified surgery widely used in studies on the hypoglycemic mechanism of gastric bypass, was employed in the present study. Our previous study showed that new biliopancreatic diversion (NBPD, Fig. 2) alone could reduce blood glucose levels, and intestinal exclusion (duodenal-jejunal exclusion, DJE, Fig. 3) alone produced no hypoglycemic effect. Also, the levels of blood bile acid changed after biliopancreatic diversion^2–4^. Therefore, we speculated that diversion of bile and pancreatic juices might affect the digestion and absorption of lipids and also the intestinal hepatic circulation of bile acids, which in turn, would reduce lipid toxicity and blood glucose levels. To investigate the effects of intestinal exclusion alone and biliopancreatic diversion alone on blood glucose levels, changes in blood glucose, blood lipids, insulin resistance (IR), and islet cell apoptosis were examined after performing DJB, NBPD, DJE, and sham operations (Fig. 4). The levels of fasting serum total bile acids and mRNA and protein expression for components in signaling pathways downstream of bile acid receptors in various organs were measured at 24 weeks postoperation. This was done to explore the role of bile acids and their pathways in the hypoglycemic mechanism of intestinal bypass, so as to further explore understand the hypoglycemic mechanism of DJB.

## Material and methods

### Animal breeding

Five-week-old specific pathogen-free, 150–200 g male Sprague Dawley rats, were purchased from Shanghai SLAC Experimental Animal Co., Ltd. (Lot number: SCXK (Hu) 2012–0002) were housed in the barrier system in the Animal Experimental Center of Fujian Medical University (5 rats per cage). The room was maintained at an ambient temperature of 22–26 °C, a humidity of 55%, and had a 12:12 h light–dark cycle. The rats were fed a normal feed (containing 6% fat), purchased from the Fuzhou Wu Experimental Animal Center, and food and water were freely available. The protocol for this study was approved by the Experimental Animal Ethics Committee of Fujian Medical University.

### Establishment of a T2DM rat model

Rats were fed a high-fat and high-sugar diet (60% normal feed, 10% lard, 10% egg yolk powder, and 20% sucrose, purchased from the Fuzhou Wu Experimental Animal Center) for 8 weeks. After overnight (12 h) fasting with fee access to water, a 1% streptozotocin (STZ) solution was intraperitoneally injected at a dose of 40 mg/kg; after which, the rats were fed the high-fat and high-sugar diet for an additional 7 days. Next, a blood sample was collected from the tail vein of each rat and its glucose level was randomly determined. Animals with a random blood glucose level ≥ 16.7 mmol/L were included in the T2DM model, and then fed a normal diet for 1 week before being used in any further experiments.

### Grouping of type 2 diabetes rat models

The model rats were randomly assigned to 4 groups (10 rats per group).

### Surgical procedures

#### Surgical procedure for NBPD

**Figure 5:**
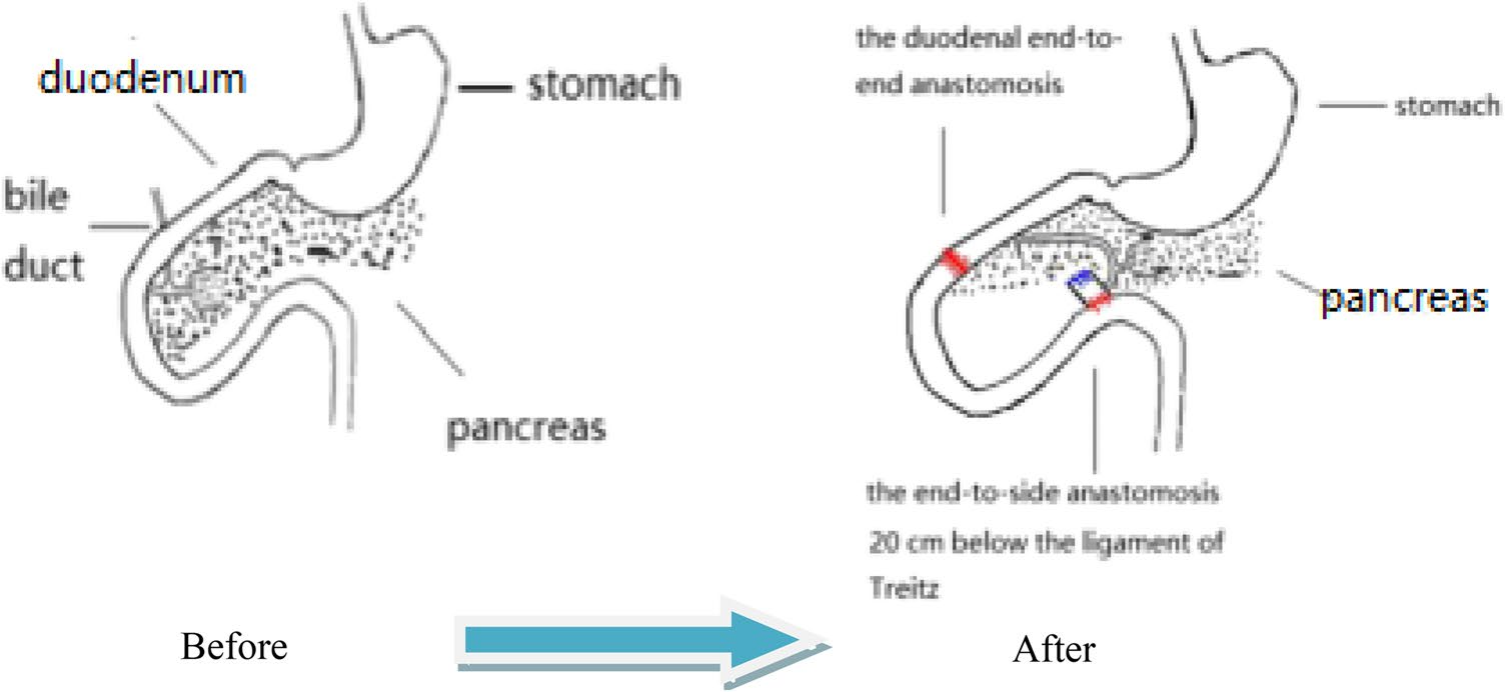
Surgical diagram for NBPD.

(1) A 1-cm intestinal segment was removed at the bile-intestine confluence. (2) The proximal end was anastomosed to the distal duodenum. (3) One end of the intestine at the bile-intestine confluence was closed, and the other end was anastomosed to the jejunum at 20 cm below the ligament of Treitz. (4) The abdominal cavity was flushed with normal saline, and the wound was closed^5^ (Fig. 5).

#### Surgical procedure for DJE

**Figure 6:**
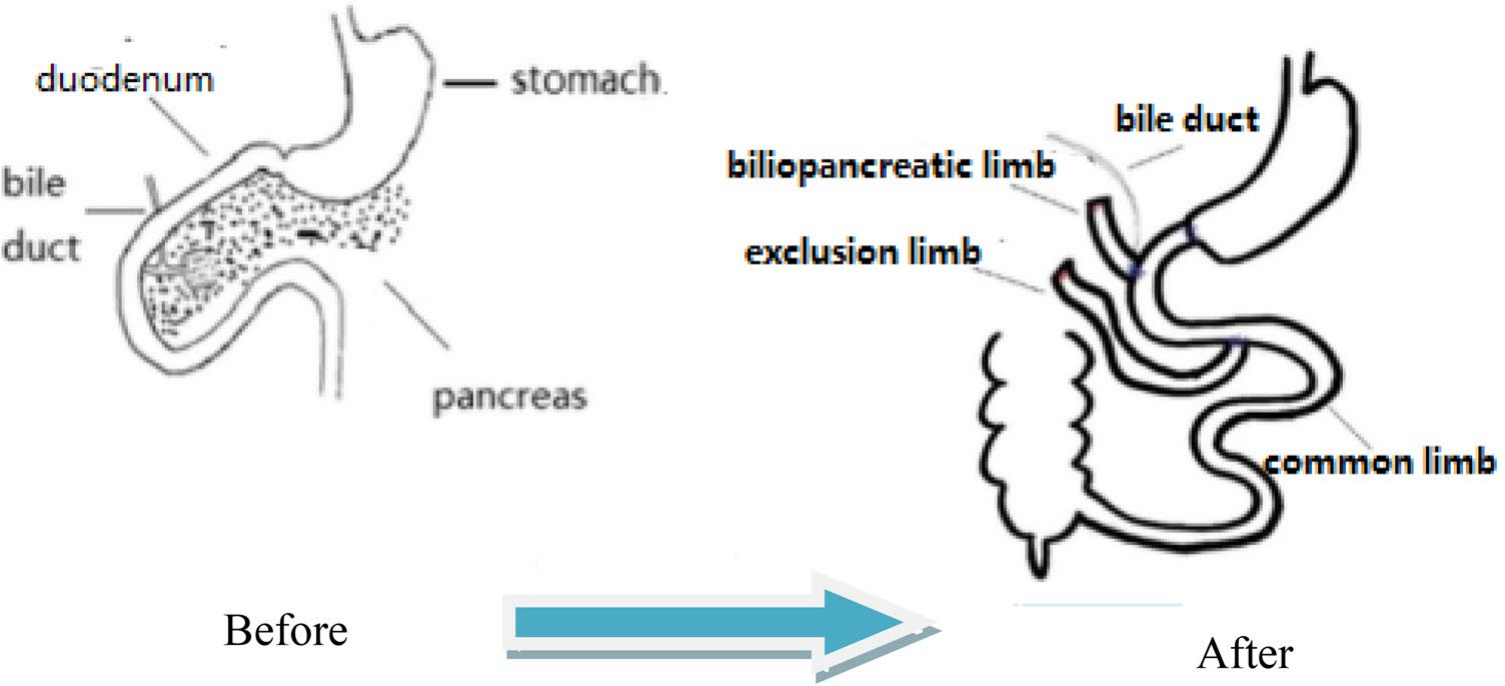
Surgical diagram for DJE.

(1) The duodenum was transected 0.5 cm below the pylorus, and its distal end was closed. (2) The jejunum was cut 10 cm below the ligament of Treitz, and the distal jejunum was anastomosed to the proximal duodenum. (3) An incision was made at 10 cm below the duodenal-jejunal anastomosis site, and the proximal jejunum was anastomosed to this anastomosis site. (4) The intestine was transected 0.5 cm below the bile intestine confluence and the distal end was closed. (5) A lateral incision was made at 5 cm below the duodenal-jejunal anastomosis site, followed by anastomosis with the intestine at the bile intestine confluence. (6) The abdominal cavity was flushed with normal saline, and the wound was closed^5^ (Fig. 6).

### Blood glucose measurements

Rats were fasted overnight for 12 h. Next, samples of tail vein blood were obtained and analyzed for their glucose levels. The rats were then fed a normal diet for 2 h; after which, samples of blood were obtained and analyzed for their 2-h postprandial glucose levels. These blood glucose measurements were made at 1 week preoperation, and again at 8 and 24 weeks postoperation, respectively, with the use of a Roche Accu-Chek blood glucose meter and test strips.

### Detection of fasting serum TG, cholesterol, and FFAs

Blood samples were collected from the iliac vein at 1 week preoperation, and again at 8 and 24 weeks postoperation. The supernatant fractions of the samples were sent to the Laboratory Department of the First Affiliated Hospital of Fujian Medical University, Aliquots of fasting serum samples were immediately analyzed for TG, cholesterol and FFAs profiles using an automatic biochemical analyzer (Hitachi, Japan). The rest samples were stored at − 80 °C for future analysis.

### Detection of insulin concentrations in rats

Blood samples were collected from the iliac vein at 1 week preoperation, and again at 8 and 24 weeks postoperation. Serum insulin concentration in response to glucose administration were measured by enzyme-linked immunosorbent methods (DRG, Germany).

### Insulin resistance index (HOMA-IR)

HOMA-IR = fasting plasma glucose (FPG, mmol/L) × fasting insulin (FINS, mIU/L)/22.5.

### Tissue sampling

Rats received an intragastric administration of 20% glucose saline solution (1 g/kg) for 2 h on week 24 postoperation, and then were sacrificed. Samples of liver and intestinal tract tissue were collected and stored in liquid nitrogen (rat samples were coded as the group number + abbreviation for the surgical method; e.g., for the 7th rat in group A using the DJB procedure, the code was A7B).

#### Western blot detection of FXR expression in the liver tissues of animals treated with different surgical procedures

Livers were homogenized in lysis buffer containing protease and phosphatase inhibitors (BestBio, Shanghai, China) and centrifuged (12,000 rpm, 10 min, 4 °C). The supernatants were extracted and the protein concentration was determined by BCA Kit (Beyotime, Shanghai, China). Equivalent amounts of samples were loaded on 8% SDS-PAGE gels (Beyotime, China) and separated by electrophoresis. Then proteins were transferred onto PVDF membrane (Millipore, USA). After blocked in 5% fat-free milk for 2 h, the membrane was divided into two parts by the help of Marker (Thermo Fisher, USA), and incubated with primary antibodies to farnesoid X receptor (FXR, 56 kDa) (ABclonal Biotech, Wuhan, China) and glyceraldehyde-3-phosphate dehydrogenase (GAPDH, 37 kDa) (CST, USA) overnight respectively, followed by incubation in horseradish peroxidase-conjugated secondary antibodies (CST, USA) for 60 min. The protein bands were visualized by ECL solution (Beyotime, Shanghai, China), and the band intensity was quantified with the ImageJ software.

#### Primer information

FXR primers:

F: TCTACAGCGAAAGTGCTGGG

R: GGAGGGCCCAATCAGATTCA

SREBP-lc primers:

F: CCATGGACGAGCTACCCTTC

R: GCCTGTGTCTCCTGTCTCAC

CYP7A1 primers:

F: AAAGCTGGCTGAGGGATTGA

R: GCCCAGAGAATAGCGAGGTG

GAPDH primers:

F: TGCACCACCAACTGCTTAGC

R: GGCATGGACTGTGGTCATGAG

Fluorescence quantitative PCR (relative quantification) was used for the following amplification steps. Initial incubation of the reaction mixture at 94 °C for 10 min, followed by 30 (FXR), 35 (SREBP-1c) or 40 (CYP7A1) cycles of 95 °C for 30 s, 60 °C for 30 s, and 72 °C for 30 s, respectively, and a final extension at 72 °C for 10 min.

#### Detection of islet cell apoptosis

Rats were sacrificed at 24 weeks postoperation and their pancreatic tissues were removed. Apoptosis of islet cells was detected by the TUNEL assay: We counted all islets identified in the tissue section, and count insulin-positive cells that are positive for TUNEL and divide by total number of beta cells^6^.

### Statistical analysis

Data for each group are presented as the mean ± standard deviation (x ± s). Inter-and intra-group statistical analyses were performed using repeated-measures multivariate analysis of variance, the F test, t test (T test for two independent samples), and one-way ANOVA of independent samples for inter-group and intra-group statistics. Relative levels of gene expression are expressed as the 2 − ΔΔCt value. All data were analyzed using IBM SPSS Statistics for Windows, Version 22 (IBM Corp., Armonk, NY, USA) and *p*-values < 0.05 were considered to be statistically significant.

### Ethic approval and consent to participate

All applicable institutional and/or national guidelines for the care and use of animals were followed. A statement confirming the study was carried out in compliance with the ARRIVE guidelines.

## Results

### Blood glucose

**Figure 7:**
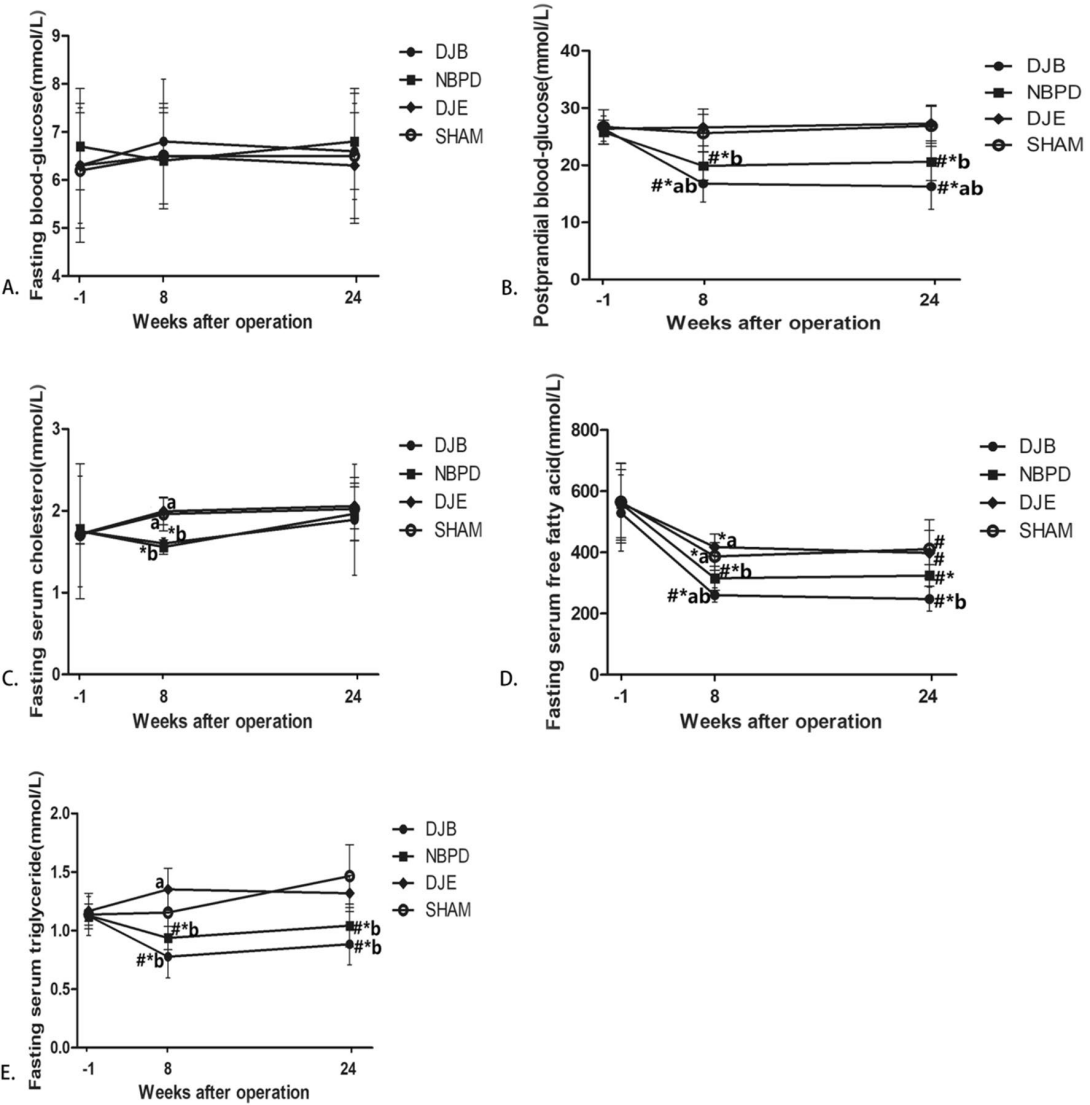
(**A**, **B**) Fasting and postprandial serum-glucose concentrations. (**C**, **D**, **E**) Fasting serum cholesterol, FFA, and TG levels. ^#^*P* < 0.05, versus Preoperation; *P < 0.05, versus the SHAM group; ^a^*P* < 0.05, versus the NBPD group; ^b^*P* < 0.05, versus the DJE group.

There were no significant differences in the fasting blood glucose levels of rats in the 4 different groups either preoperation or postoperation (Fig. 7A). However, the 2-h postprandial blood glucose levels in the DJB and NBPD groups were significantly lower than those in the SHAM and DJE groups at 8 and 24 weeks postoperation and also at 1 week preoperation (*p* < 0.05) (Fig. 7B).

### Fasting serum cholesterol, FFAs, and TGs

While the fasting serum cholesterol levels in the DJB and NBPD groups were significantly lower than those in the SHAM and DJE groups at 8 weeks postoperation (*p* < 0.05), no significant difference in fasting serum cholesterol levels was found among the 4 groups at 24 weeks postoperation (Fig. 7C). However, the fasting serum FFA levels in the DJB and NBPD groups were significantly lower than those in the SHAM and DJE groups at 8 and 24 weeks postoperation (*p* < 0.05) (Fig. 7D). The fasting serum TG levels in the DJB and NBPD groups were significantly lower than those in the SHAM and DJE groups at 8 and 24 weeks postoperation and also at 1 week preoperation (*p* < 0.05). The fasting TG levels in the DJE group were significantly higher than those in the NBPD group at 8 weeks postoperation (*p* < 0.05). No differences in other measurements were found when compared with the SHAM group (Fig. 7E).

### Fasting serum insulin and HOMA-IR

**Figure 8:**
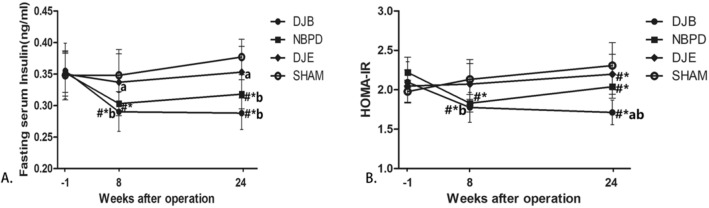
(**A**, **B**) Fasting serum insulin concentrations and HOMA-IR. ^#^*P* < 0.05, versus preoperation; *P < 0.05, versus the SHAM group; ^a^*P* < 0.05, versus the NBPD group; ^b^*P* < 0.05, versus the DJE group.

Serum insulin levels in the DJB and NBPD groups were significantly lower than those in the SHAM and DJE groups at 8 and 24 weeks postoperation and also at 1 week preoperation (*p* < 0.05) (Fig. 8A). The HOMA-IR values in the DJB and NBPD groups were significantly lower than those in the SHAM group at 8 and 24 weeks postoperation and also at 1 week preoperation. Furthermore, the HOMA-IR values in the DJB group were significantly lower than those in the DJE group at both 8 and 24 weeks postoperation (*p* < 0.05) (Fig. 8B).

### Apoptosis rate of islet cells

**Figure 9:**
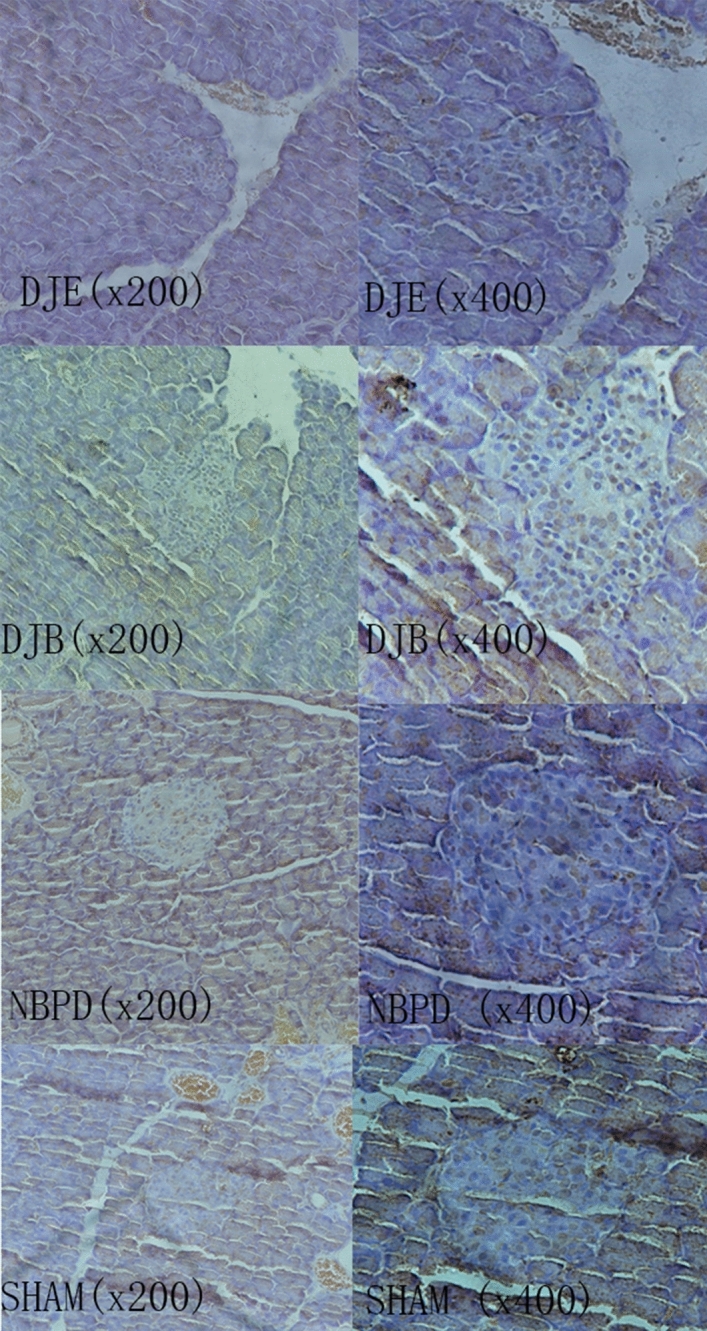
Apoptosis of islet cells as determined by the Tunel assay.

The apoptosis rates of islet cells in the DJB and NBPD groups were significantly lower than that in the SHAM group (*p* < 0.05), and no significant difference was found between the apoptosis rates in the DJE group and SHAM group (Table 1) (Fig. 9).

**Table 1:** Apoptosis rate of islet cells in rats (*x* ± s).

	DJB group	NBPD group	DJE group	SHAM group
Apoptosis rate	0.314 ± 0.076*^b^	0.339 ± 0.072*^b^	0.399 ± 0.058^a^	0.408 ± 0.053^a^

*P < 0.05, versus the SHAM group.

^a^*P* < 0.05, versus the NBPD group.

^b^*P* < 0.05, versus the DJE group.

### PCR, qPCR and western-blot results

**Figure 10:**
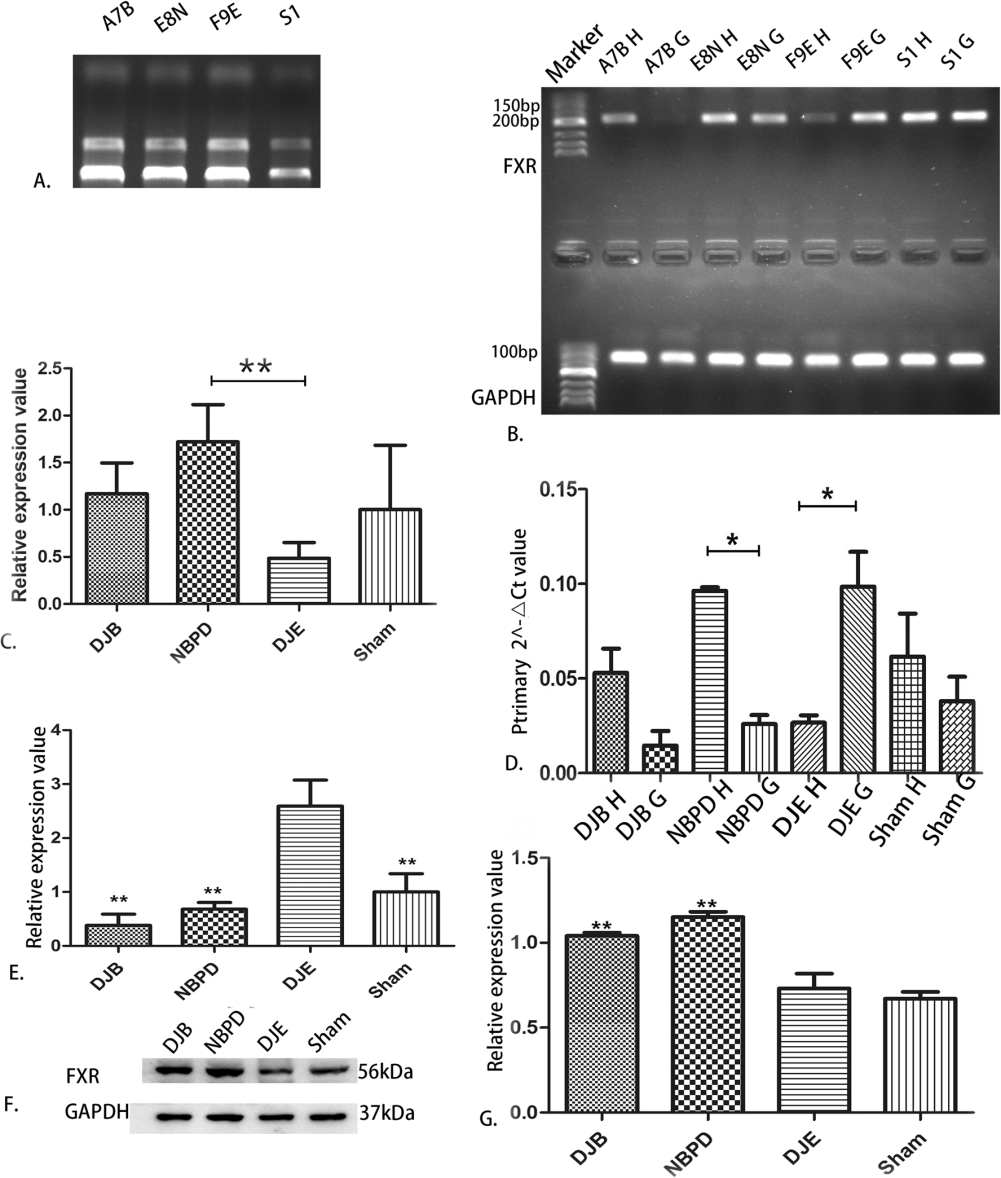
(**A**) Quality verification of RNA samples by agarose electrophoresis. (**B**) Electrophoresis of representative qPCR products of the *FXR* and *GAPDH* genes (internal reference). (**C**) Relative expression levels of liver FXR mRNA in animals treated via different operations. (**D**) Original 2 − ΔΔCt values of *FXR* gene qPCR products from randomly selected samples (marker: Takara DL500). (**E**) Relative expression levels of intestinal FXR mRNA in animals treated via different operations. (**F**) Electrophoresis of liver FXR protein obtained from animals treated via different operations. (**G**) Expression of FXR protein in the liver and gall of animals treated via different operations.

**Figure 11:**
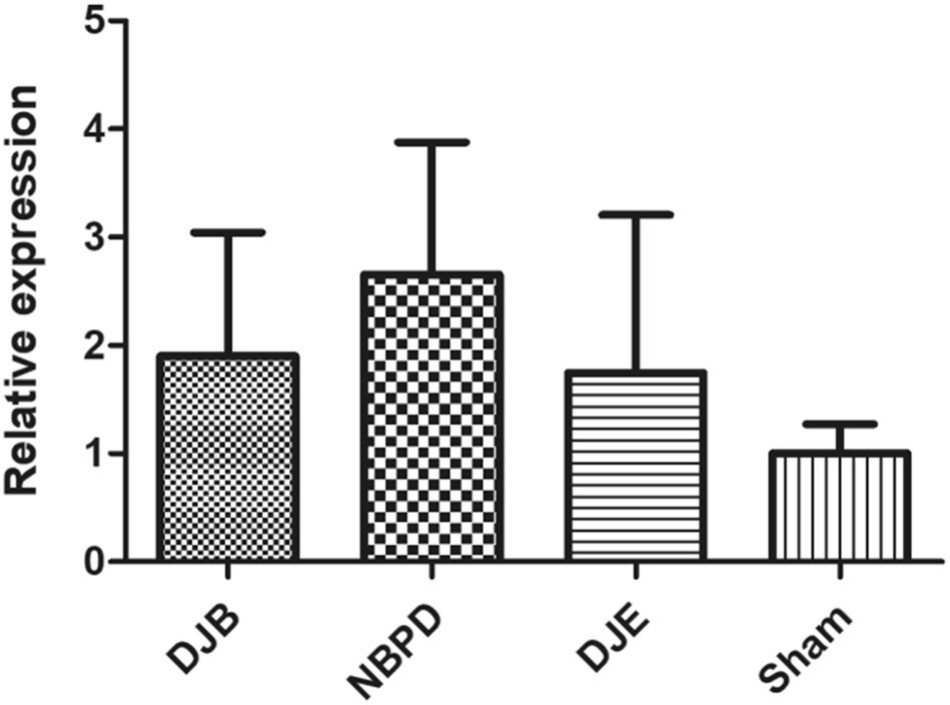
Relative expression of SREBP-1c mRNA.

**Figure 12:**
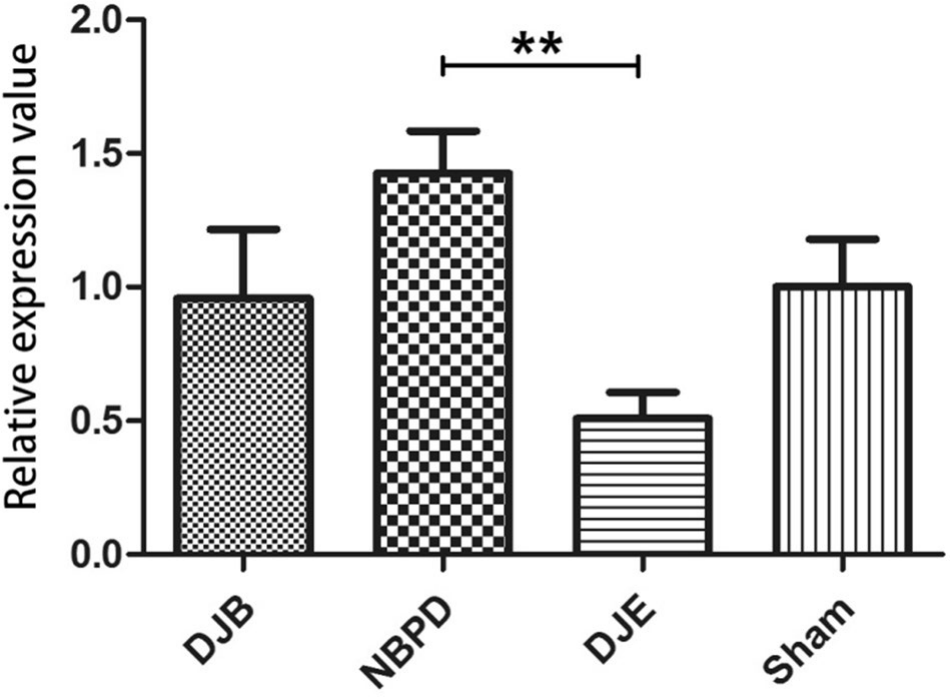
Relative expression of liver CYP7A1 mRNA in rats treated via different operations.

**Figure 13:**
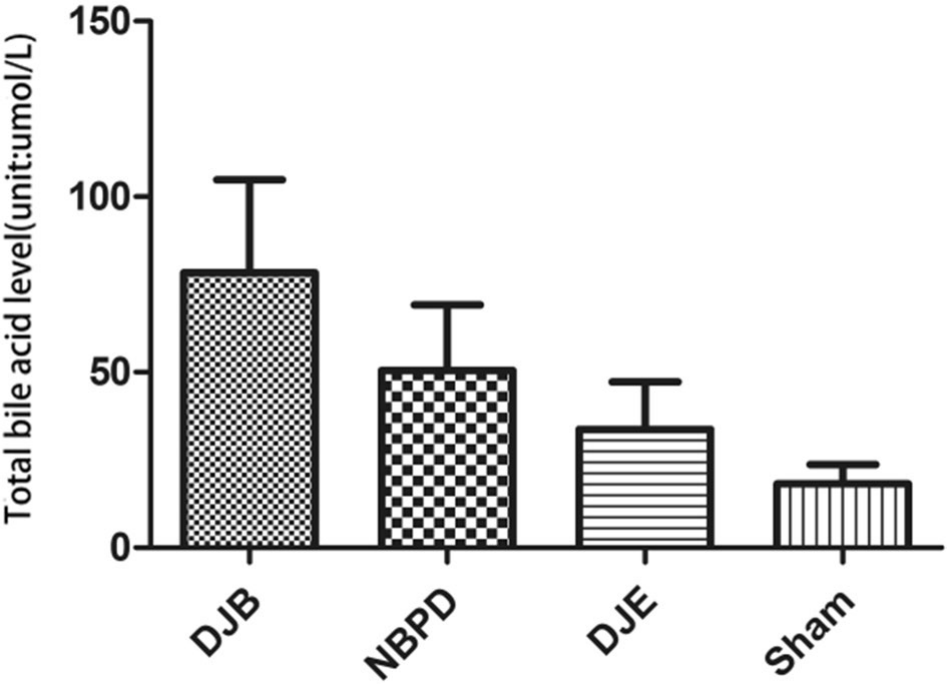
Total fasting serum bile acid concentrations in rats treated via different operations.

1. RNA was not degraded, and its quality was high (Fig. 10A).
2. A comparison of the relative expression of liver FXR mRNA(Relative expression is presented as the difference in gene expression (expressed by multiples) between the experimental group and the control group i.e., the 2−ΔΔCt value.) in animals treated via different operations (Fig. 10C) showed that the levels of FXR mRNA in the DJB group were significantly higher than those in the DJE group (*p* < 0.05), but did not significantly differ from those in the NBPD and SHAM groups; furthermore, FXR expression in the NBPD group was significantly higher than that in the DJE group (*p* < 0.01).
3. A comparison of the relative expression of intestinal FXR mRNA in animals treated via different operations (Fig. 10E) showed that the levels of FXR mRNA in the DJE group were significantly higher than those in the DJB, NBPD, and SHAM groups (*p* < 0.01).
4. Results of electrophoresis of qPCR products produced by the *FXR* and *GAPDH* genes in animals treated via different operations (Fig. 10B) were consistent with the original 2 − ΔΔCt values of the *FXR* gene qPCR products for each corresponding operation (Fig. 10 D).
5. An electrophoresis of liver FXR protein (Fig. 10 F) and an analysis of liver FXR protein expression in animals treated via different operations (Fig. 10G) showed that the levels of liver FXR protein in the NBPD and DJB groups were significantly higher than those in the DJE and SHAM groups (*p* < 0.01). (The blots were cut prior to hybridisation with antibodies, the 37 kDa and 56 kDa were closely, so the membrane should have been a whole membrane before we divided it, and it was area of blank above them)
6. A comparison of the relative expression of liver SREBP-1c mRNA in animals treated via different operations (Fig. 11) showed no significant difference in SREBP-1c mRNA expression among the different operation groups (*p* > 0.05).
7. A comparison of relative expression of liver CYP7A1 mRNA in animals treated via different operations (Fig. 12) showed that the levels of CYP7A1 mRNA in the NBPD group were significantly higher than those in the DJE group (*p* < 0.05).
8. A comparison of fasting serum bile acid concentrations in rats treated via different operations (Fig. 13) showed no significant difference among the groups (*p* > 0.05).

## Discussion

DJB is a surgical procedure commonly used to investigate the mechanism of how surgery affects diabetes. This procedure preserves the original biliopancreatic diversion of a gastric bypass and also the duodenal- lower jejunal exclusion. NBPD with biliopancreatic diversion alone and DJE with duodenal- jejunum exclusion alone were used in the present study to further investigate the hypoglycemic mechanism of DJB. Our previous studies showed that blood glucose levels were improved in both the DJB and NBPD treatment groups, but not in a DJE treatment group. Our present study showed that 2-h postprandial blood glucose levels in the DJB and NBPD groups as determined at 8 and 24 weeks postoperation were lower than those in the DJE and SHAM groups (*p* < 0.05) and significantly lower than those at 1 week preoperation (*p* < 0.05). The 2-h postprandial blood glucose levels in the DJE and SHAM groups at 8 and 24 weeks postoperation were not significantly different from those detected at 1 week preoperation. These results showed that the hypoglycemic effects of DJB and NBPD were stable and reliable, and that DJE or a sham operation could not reduce blood glucose levels.

Bile and pancreatic fluid are important digestive juices. Bile acid is the principal component of bile, and also an important ligand for the bile acid pathway. Our previous studies found that the postoperative blood bile acid levels in the DJB and NBPD groups were higher than those in SHAM and DJE groups^3^. Our present study showed that the levels of serum total bile acids at 24 weeks postoperation in the DJB, NBPD, DJE, and SHAM groups were 78.32 ± 26.51, 50.45 ± 18.75, 33.63 ± 13.58, and 18.13 ± 5.51 μmol/L, respectively, and the trend was consistent with findings in our previous studies; however, the differences among these groups were not statistically significant (*p* > 0.05). Reactive components of the bile acid signaling pathway, including the farnesoid X receptor (FXR, also known as the bile acid receptor) and the small heterodimer partner (SHP), are distributed throughout the body. These components mediate the expression of lipid metabolism-related genes to inhibit the de novo synthesis of fatty acids in the liver, which can be affected by an altered bile acid concentration. Previous studies^5^ reported that secretion of fibroblast growth factor 15 (FGF15) was reduced if intestinal FXR was not activated or was inhibited. Insufficient FXR activity resulted in the reduced inhibition of CYP7A1 (a rate-limiting enzyme for bile acid synthesis) and a subsequent expansion of the bile acid pool to effectively reduce body weight and blood glucose levels. However, activation of lipid metabolism pathways in the liver, such as the FXR-SHP-Srebp-1c pathway^7,8^, depends on an expansion of serum and liver bile acid pools, and plays a major role in inhibiting the synthesis of cholesterol lipids, which can alleviate insulin resistance in liver and islet cells^9^. Moreover, CYP7A1 activity in the liver is regulated by negative feedback provided by intestinal FXR, and this regulation constitutes a bile acid "liver-intestinal negative feedback mechanism." Changes in any component of the feedback loop may alter the other components. (Bile acid feedback loop: Intestinal FXR activation↑ → FGF15 (fibroblast growth factor 15,with the help of DEIT1 gene) is produced↑ → The expression of liver CYP7A1 down-regulates↓ → Serum bile acid down-regulates↓ → Bile acid pool decreases↓ → Liver FXR/ Intestinal FXR inhibition↓ → The level of FGF15 shrink↓ → CYP7A1 activation↑ → The bile acid pool is enlarged↑ → Intestinal FXR activation↑).

Our present study revealed that FXR mRNA was expressed at significantly higher levels in the livers of rats in the DJB and NBPD groups than in the livers of rats in the DJE group. Furthermore, the levels of FXR mRNA in the DJB and NBPD groups were higher than those in the SHAM group; however, that difference was not statistically significant. A subsequent test for FXR protein expression revealed that the FXR levels in the DJB and NBPD groups were significantly higher than those in the DJE and SHAM groups (*p* < 0.05). In the intestine, the levels of FXR mRNA expression in the DJE group were significantly higher than those in the DJB, NBPD, and SHAM groups, which supported the findings mentioned above. The mean level of CYP7A1 mRNA expression in the livers of rats in the NBPD group (1.426 ± 0.159) was significantly higher than that in the DJE group (0.510 ± 0.098, *p* < 0.05); however, there was no significant difference in CYP7A1 mRNA expression between the DJB group (0.957 ± 0.259) and SHAM group (1.000 ± 0.180) (*p* > 0.05), indicating that the amount of secreted of bile acids had increased in the NBPD group. Therefore, we believe that elevated serum bile acid levels had up-regulated the expression of liver FXR and down-regulated the expression of intestinal FXR. An up-regulation of FXR expression in the liver could reduce lipid deposition and lipotoxicity in the liver and exert a hypoglycemic effect by activating lipid metabolism pathways.

Studies have suggested that the FXR-SHP-Srebp-1c pathway^7,8^ may play a major role in the hypoglycemic effect of a surgical procedure. Our present study detected expression of Srebp-1c downstream of FXR in the liver, and our results showed no statistically significant difference in Srebp-1c mRNA expression among the different groups. Furthermore, the fasting serum cholesterol levels before and after surgery in all surgical rats (Fig. 7C) were not significantly different at 24 weeks postoperation when compared with those at 1 week preoperation, and also did not significantly vary among the different groups (*p* > 0.05).

Recent studies have focused on interactions that occur between the targets of hypoglycemic drugs; i.e., transcription factor YY1 and liver FXR^10^. It is believed that YY1 can inhibit transcription of the liver *FXR* gene and activate Srebp-1c. In addition, YY1 may also up-regulate the expression of lipogenesis-related genes (such as the fatty acid synthase gene)^10,11^, resulting in excessive deposition of TGs and lipotoxicity in the liver. The results of our present study (Fig. 7E) showed that the fasting TG levels in the DJB and NBPD groups at 24 weeks postoperation were significantly lower than those at 1 week preoperation (p < 0.05), and the fasting TG levels in the DJB and NBPD groups at 24 weeks postoperation were significantly lower than those in the DJE and SHAM groups (*p* < 0.05). These finding indicated that the operation group with high liver FXR expression, however, has a low TG level. The ectopic deposition of TGs and FFAs plays an important role in the process of lipotoxicity leading to T2DM. Studies have shown that TGs and FFAs are closely related to the development and progression of diabetes, and may induce insulin resistance in organs and tissues^12–14^, and apoptosis in islet cells^15^,while a decreased level of FFAs may help to alleviate insulin resistance^16^. In the present study, the HOMA-IR values decreased in both the intestinal bypass and modified biliopancreatic diversion groups, while no significant change was observed in the intestinal exclusion group and sham operation group. These results suggest that the intestinal bypass surgery had reduced serum FFA levels and lipotoxicity via biliopancreatic diversion, and thereby alleviated insulin resistance and inhibited islet cell apoptosis.

In summary, biliopancreatic diversion primarily accounts for the hypoglycemic mechanism of intestinal bypass surgery. Biliopancreatic diversion can reduce lipotoxicity and improve the sensitivity of cells to insulin in T2DM rats by affecting bile acid pathways. These effects serve to alleviate insulin resistance and inhibit islet cell apoptosis, which eventually produces a hypoglycemic effect.

## Conclusion

DJB postoperatively up-regulates the expression of bile acid receptors in the liver and down-regulates those receptors in the intestinal tract via biliopancreatic diversion. These changes reduce TG levels, and subsequently the lipotoxic effects of TGs and FFAs on islet cells to produce a hypoglycemic effect.

## Supplementary Information

Supplementary Information 1.

Supplementary Figure 1.

Supplementary Figure 2.

Supplementary Figure 3.

Supplementary Figure 4.

Supplementary Figure 5.

Supplementary Figure 6.

Supplementary Figure 7.

Supplementary Figure 8.

Supplementary Figure 9.

Supplementary Figure 10.

Supplementary Figure 11.

## Data Availability

They have been attached to the submission.

## References

[1] Association AD. 6. Obesity management for the treatment of type 2 diabetes. Diabetes Care **39**, S47. 10.2337/dc16-S009 (2016). 10.2337/dc16-S009 26696681

[2] Weng S, Zhang B, Xu C (2016). Influence of new modified biliopancreatic diversion on blood glucose in GK rats for a long period. World Chin. J. Digestol..

[3] Weng SG, Zhang B, Wang X (2017). Effects of duodenal-jejunal exclusion and new bilio-pancreatic diversion on blood glucose in rats with type 2 diabetes mellitus. Obes. Surg..

[4] Weng S, Zhang B, Xu C (2017). Influence of new modified biliopancreatic diversion on blood glucose and lipids in GK rats. Obes. Surg..

[5] Kim I, Ahn SH, Inagaki T (2007). Differential regulation of bile acid homeostasis by the farnesoid X receptor in liver and intestine. J. Lip. Res..

[6] Téllez N, Montanya E (2020). Determining beta cell mass, apoptosis, proliferation, and individual beta cell size in pancreatic sections. Methods Mol. Biol..

[7] Han H, Wang L, Du H (2015). Expedited biliopancreatic juice flow to the distal gut benefits the diabetes control after duodenal-jejunal bypass. Obes. Surg..

[8] Watanabe M, Houten SM, Wang L (2004). Bile acids lower triglyceride levels via a pathway involving FXR, SHP, and SREBP-1c. J. Clin. Investig..

[9] Petersen KF, Dufour S, Befroy D (2005). Reversal of nonalcoholic hepatic steatosis, hepatic insulin resistance, and hyperglycemia by moderate weight reduction inpatients with type 2 diabetes. Diabetes.

[10] Lu Y, Ma Z, Zhang Z, Xiong X (2014). Yin Yang 1 promotes hepatic steatosis through repression of farnesoid X receptor in obese mice. Gut.

[11] Rajendra R, Chandmhasa Y, Xiong D (2008). SREBPs: the crossroads of physiological and pathological lipid homeostasis. Trends Endocrinol. Metabol..

[12] McGarry JD (2002). dysregulation of fatty acid metabolism in the etiology of type 2 diabetes. Diabetes.

[13] Boden G (1997). Role of fatty acids in the pathogenesis of insulin resistance and NIDDM. Diabetes.

[14] Arner P, Rydén M (2015). Fatty acids, obesity and insulin resistance. Obes. Facts.

[15] Natalicchio A, Labarbuta R, Tortosa F (2013). Exendin-4 protects pancreatic beta cells from palmitate-induced apoptosis by interfering with GPR40 and the MKK4/7 stress kinase signalling pathway. Diabetologia.

[16] Makimura H, Stanley TL, Suresh C (2016). Metabolic effects of long-term reduction in free fatty acids with acipimox in obesity: A randomized trial. J. Clin. Endocrinol. Metab..

