# Supplementary material for: Hypoglycemic mechanism of intestinal bypass surgery in type 2 diabetic rats
Source: Sci Rep. 2021 Nov 3;11:21596. doi: 10.1038/s41598-021-98714-1 (PMC8566479; doi:10.1038/s41598-021-98714-1)
Supplement: Supplementary file 1 — Supplementary Information 1. [file 41598_2021_98714_MOESM1_ESM.docx]

**Hypoglycemic Mechanism of Intestinal Bypass Surgery in Type 2 Diabetic Rats**

Siqi Xie, MM^a,#^, MingChang Wang, MM ^b,#^, Bin Zhang, MM^a^, ShanGeng Weng, MD^a,*^.

^a^ Hepatopancreatobiliary Surgery Department, the First Affiliated Hospital of Fujian Medical University, National Abdominal Surgery Institute of Fujian, Fuzhou City, Fujian, PR China

^b^ Department of Intensive Care Unit, The Second Affiliated Hospital of Fujian Medical University, No. 34, North Zhongshan Road, Licheng District, Quanzhou City, Fujian, PR China

**^a^Siqi Xie, MM,** Hepatopancreatobiliary Surgery Department, the First Affiliated Hospital of Fujian Medical University, No 20 Chazhong Road, Fuzhou City, Fujian, PR China.

**^b^MingChang Wang, MM,** Department of Intensive Care Unit, The Second Affiliated Hospital of Fujian Medical University, No. 34, North Zhongshan Road, Licheng District, Quanzhou City, Fujian, PR China

**^a^Bin Zhang, MM,** Hepatopancreatobiliary Surgery Department, the First Affiliated Hospital of Fujian Medical University, No 20 Chazhong Road, Fuzhou City, Fujian, PR China.

**^a^ShanGeng Weng, MD,** Hepatopancreatobiliary Surgery Department, the First Affiliated Hospital of Fujian Medical University, No 20 Chazhong Road, Fuzhou City, Fujian, PR China.

**^#^** Siqi Xie and Mingchang Wang contributed equally to this article.

*** Corresponding author：**ShanGeng Weng, Hepatopancreatobiliary Surgery Department, the First Affiliated Hospital of Fujian Medical University, No 20 Chazhong Road, Fuzhou City, Fujian, PR China. Tel.: t86 13788877012; fax: t86 059183356180.

E-mail address: shangeng@sina.com (S. Weng).


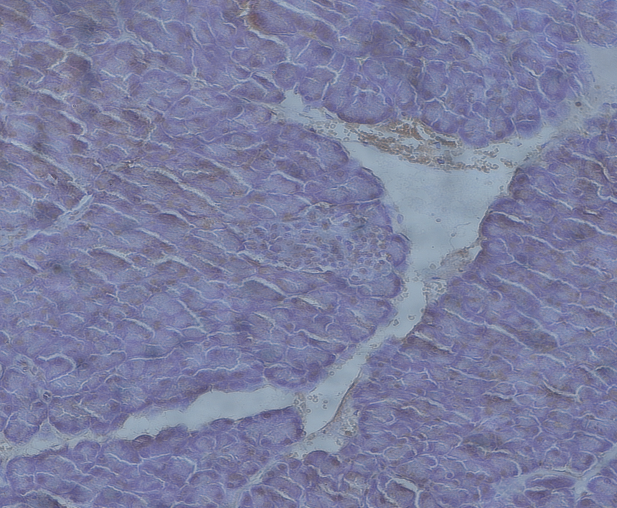


**Fig 9.** Apoptosis of islet cells in DJE group under the “x200” field of view

**Supplementary Fig 9 DJE(x200).** The micrographs of DJE group under the “x200” field of view.


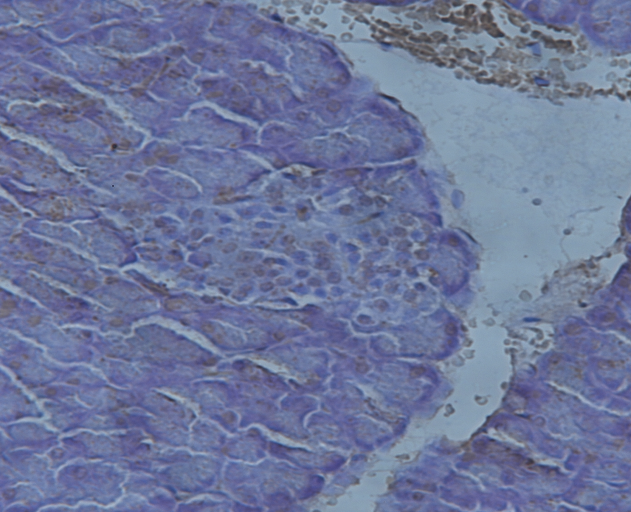


**Fig 9.** Apoptosis of islet cells in DJE group under the “x400” field of view

**Supplementary Fig 9 DJE(x400).** The micrographs of DJE group under the “x400” field of view.


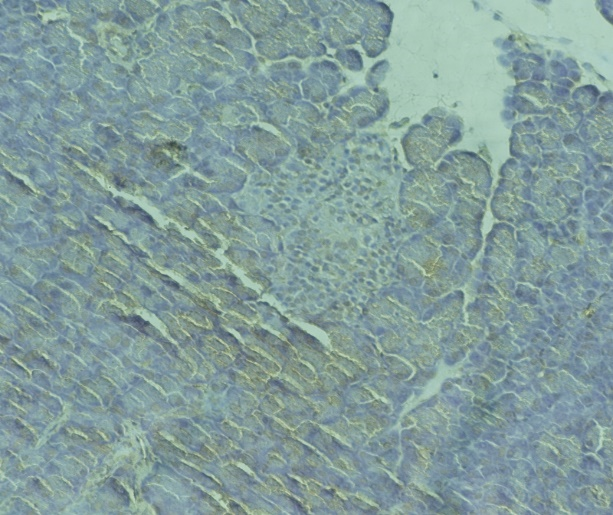


**Fig 9.** Apoptosis of islet cells in DJB group under the “x200” field of view

**Supplementary Fig 9 DJB(x200).** The micrographs of DJB group under the “x200” field of view.


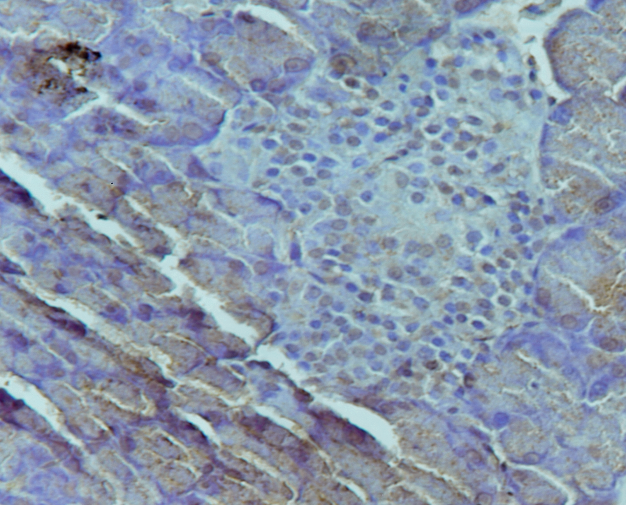


**Fig 9.** Apoptosis of islet cells in DJB group under the “x400” field of view

**Supplementary Fig 9 DJB(x400).** The micrographs of DJB group under the “x400” field of view.


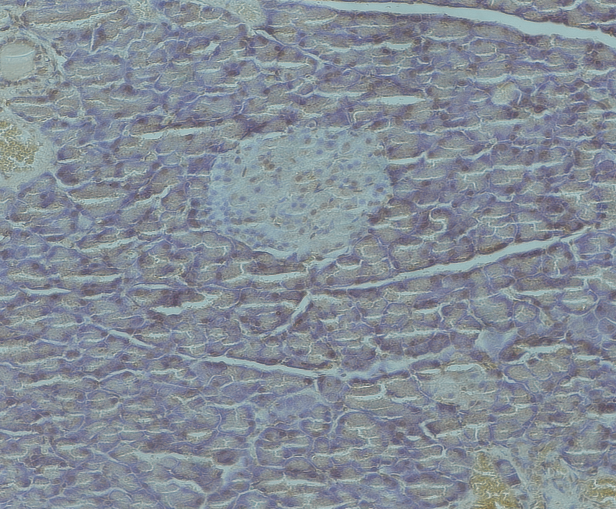


**Fig 9.** Apoptosis of islet cells in NBPD group under the “x200” field of view

**Supplementary Fig 9 NBPD(x200).** The micrographs of NBPD group under the “x200” field of view.


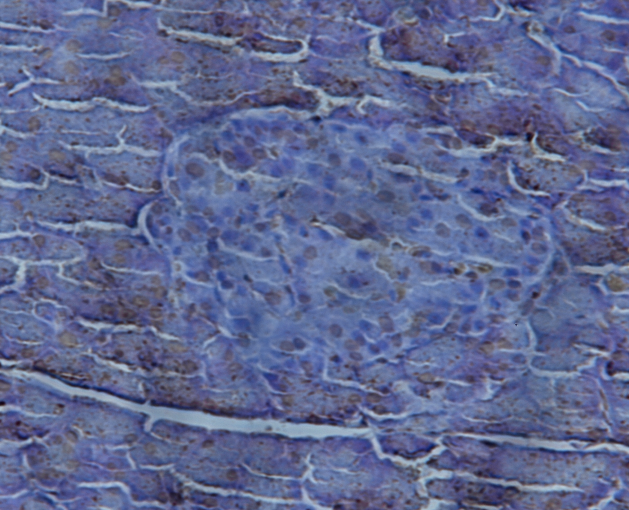


**Fig 9.** Apoptosis of islet cells in NBPD group under the “x400” field of view

**Supplementary Fig 9 NBPD(x400).** The micrographs of NBPD group under the “x400” field of view.


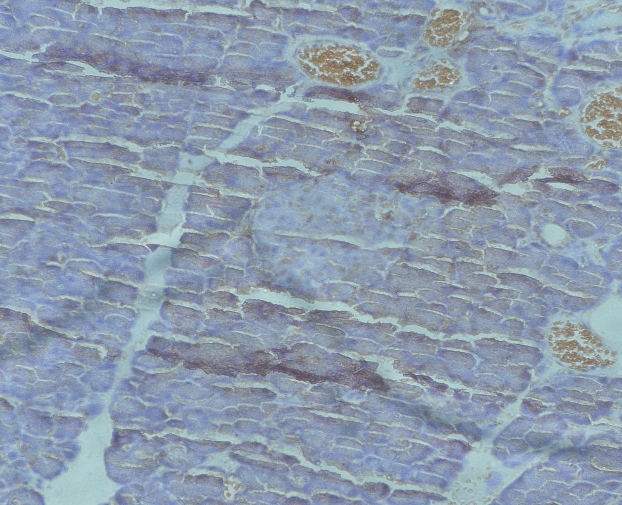


**Fig 9.** Apoptosis of islet cells in SHAM group under the “x200” field of view

**Supplementary Fig 9 SHAM(x200).** The micrographs of SHAM group under the “x200” field of view.


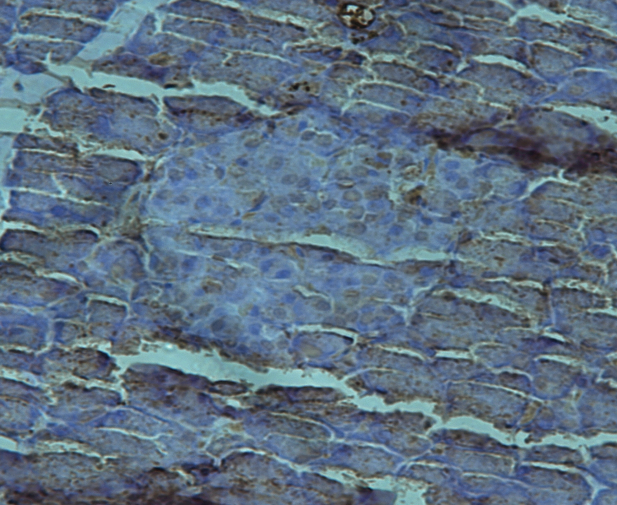


**Fig 9.** Apoptosis of islet cells in SHAM group under the “x400” field of view

**Supplementary Fig 9 SHAM(x400).** The micrographs of SHAM group under the “x400” field of view.


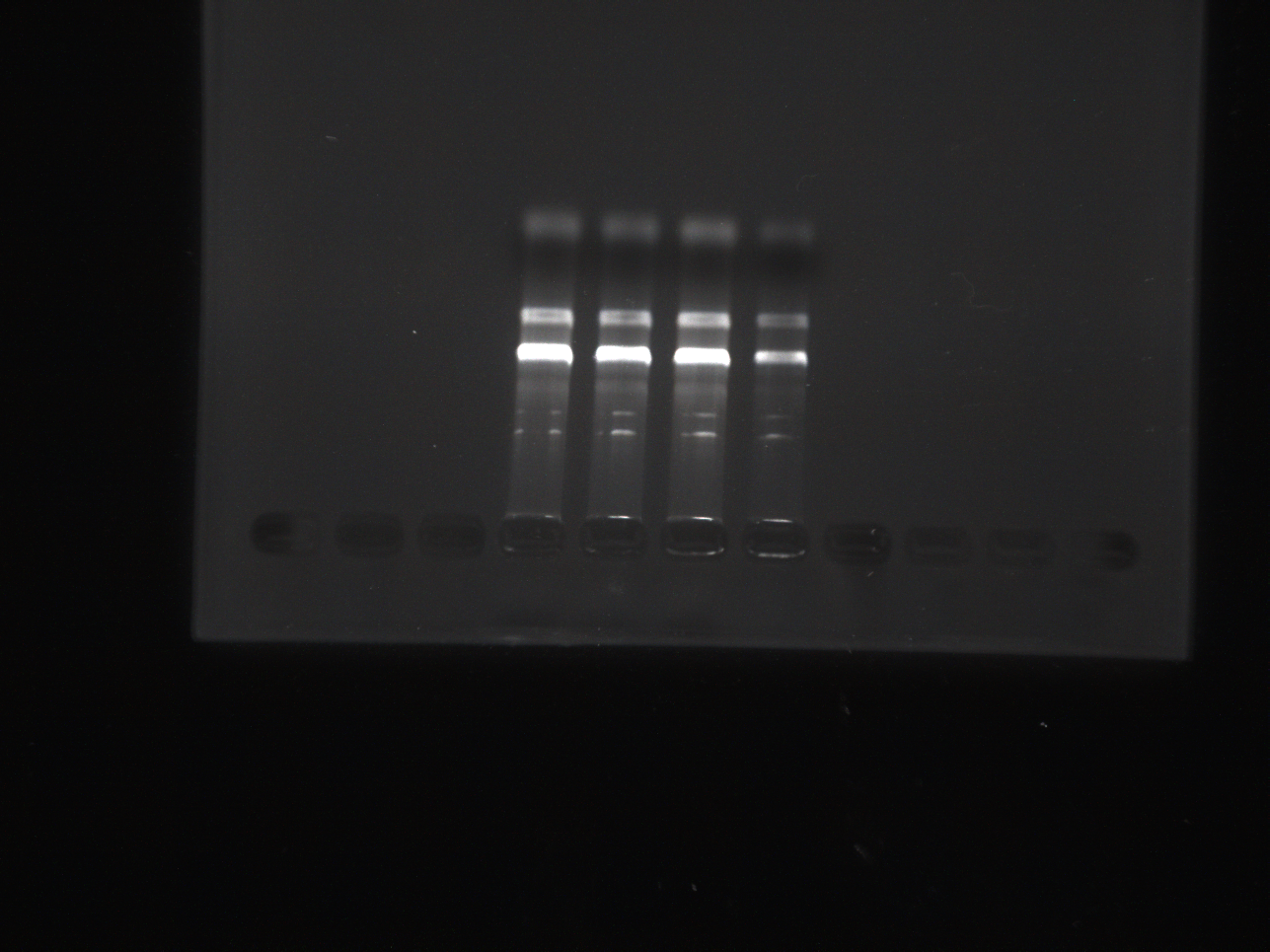


**Fig 10.A.** Quality verification of RNA samples by agarose electrophoresis.

**Supplementary Fig 10.A.** Quality verification of RNA samples by agarose electrophoresis.


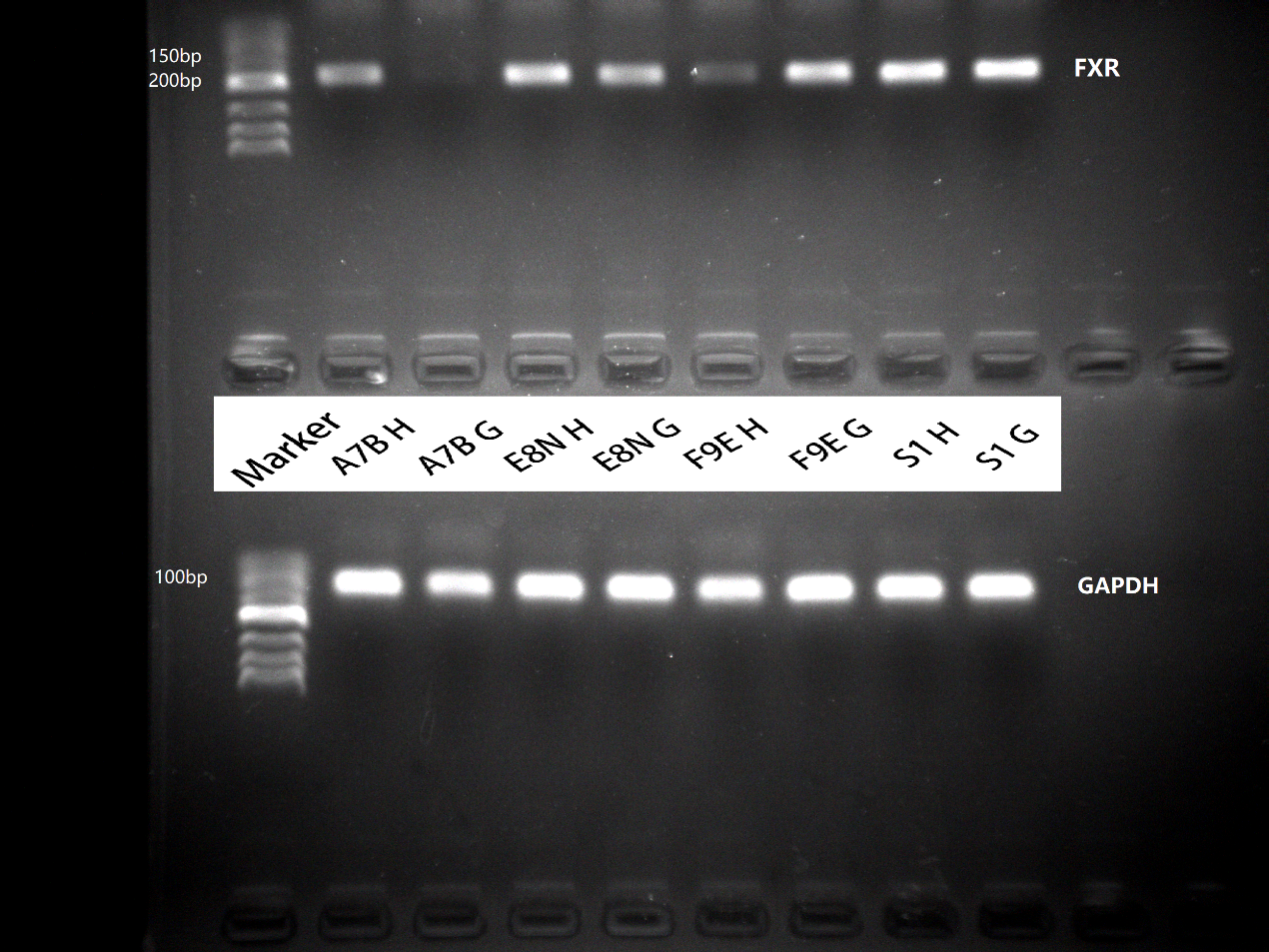


**Fig 10.B.** Electrophoresis of representative qPCR products of the *FXR* and *GAPDH* genes (internal reference)

**Supplementary Fig 10.B.** Electrophoresis of representative qPCR products of the *FXR* and *GAPDH* genes (internal reference) (marker: Takara DL500, Japan)


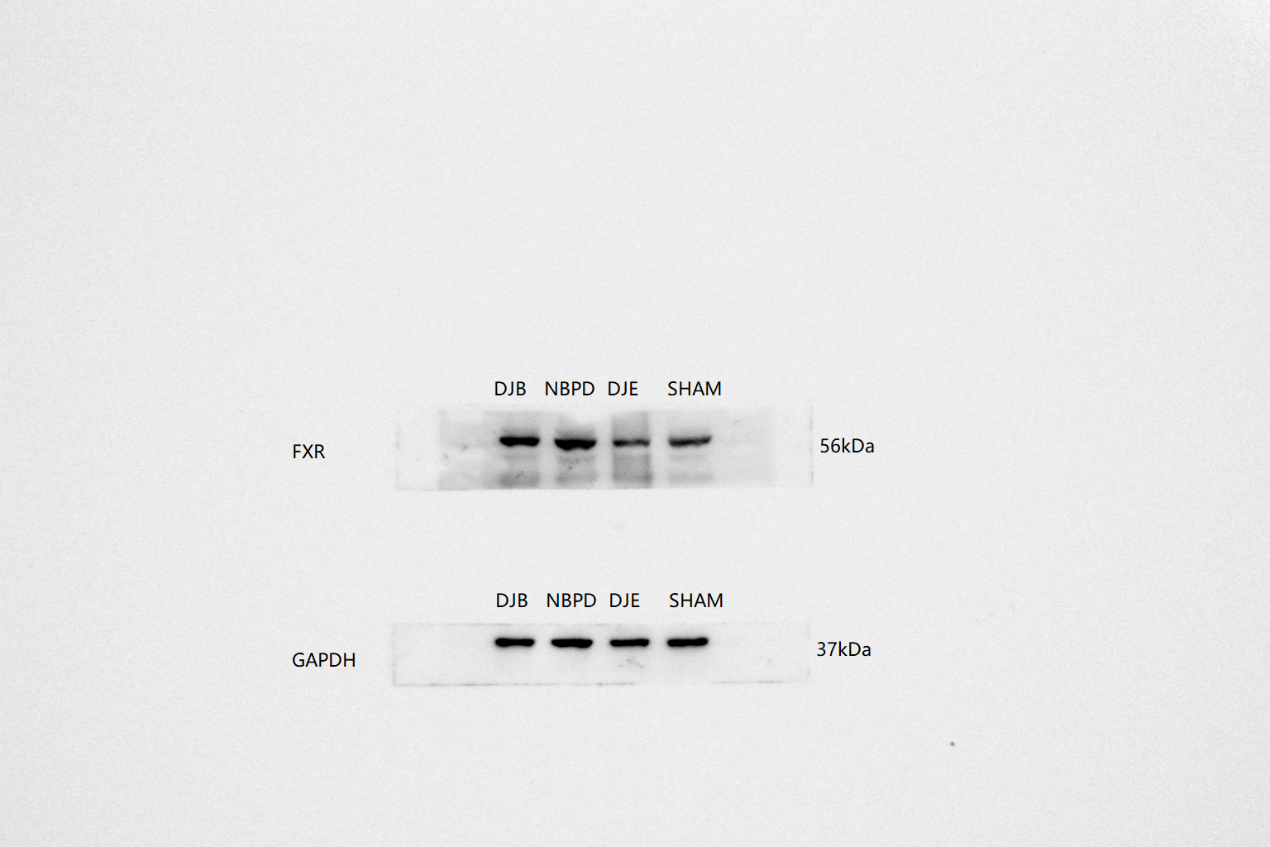


**Fig 10.F.** Electrophoresis of liver FXR protein obtained from animals treated via different operations.

**Supplementary Fig 10.F.** Electrophoresis of liver FXR protein obtained from animals treated via different operations.
