# Supplementary figures and images for: Hypoglycemic mechanism of intestinal bypass surgery in type 2 diabetic rats
Source: Sci Rep. 2021 Nov 3;11:21596. doi: 10.1038/s41598-021-98714-1 (PMC8566479; doi:10.1038/s41598-021-98714-1)

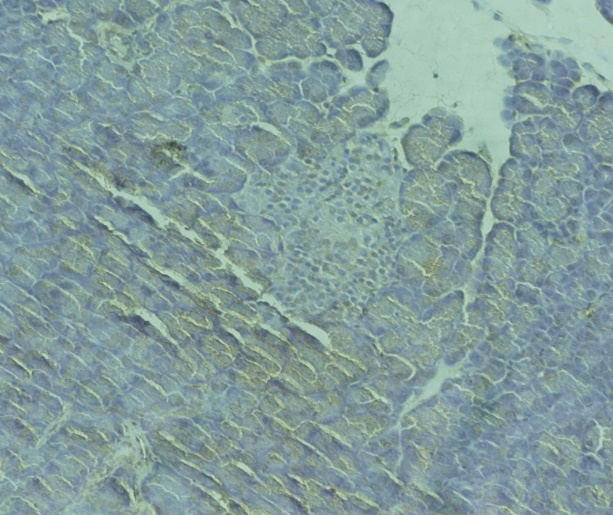

Supplement: Supplementary file 2 — Supplementary Figure 1. [file 41598_2021_98714_MOESM2_ESM.tif]

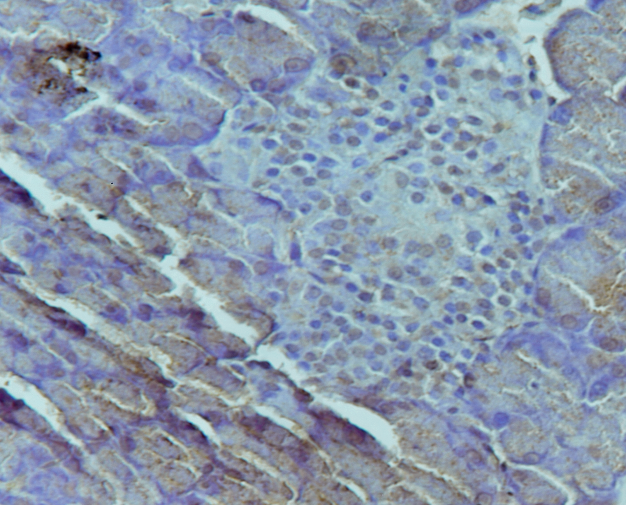

Supplement: Supplementary file 3 — Supplementary Figure 2. [file 41598_2021_98714_MOESM3_ESM.tif]

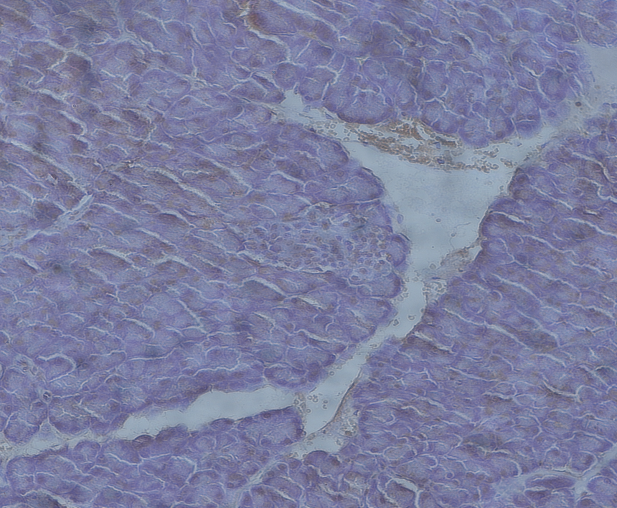

Supplement: Supplementary file 4 — Supplementary Figure 3. [file 41598_2021_98714_MOESM4_ESM.tif]

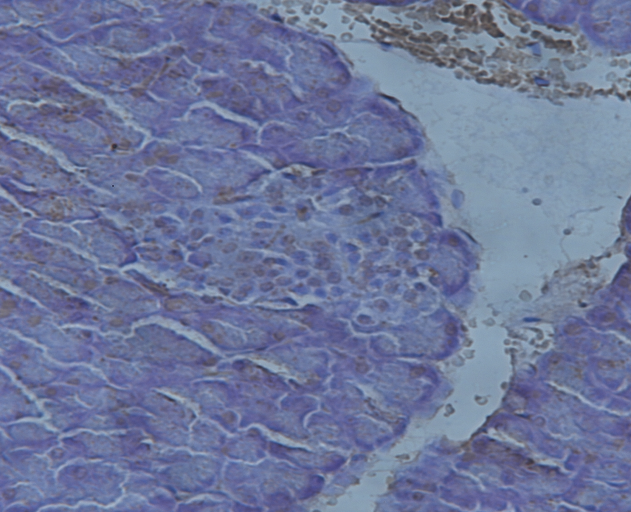

Supplement: Supplementary file 5 — Supplementary Figure 4. [file 41598_2021_98714_MOESM5_ESM.tif]

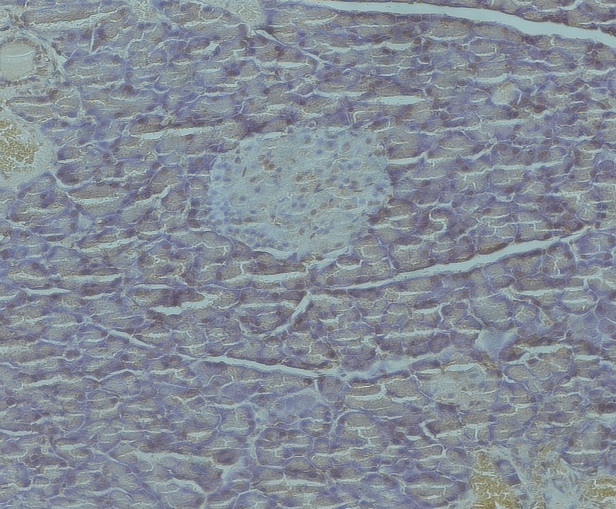

Supplement: Supplementary file 6 — Supplementary Figure 5. [file 41598_2021_98714_MOESM6_ESM.tif]

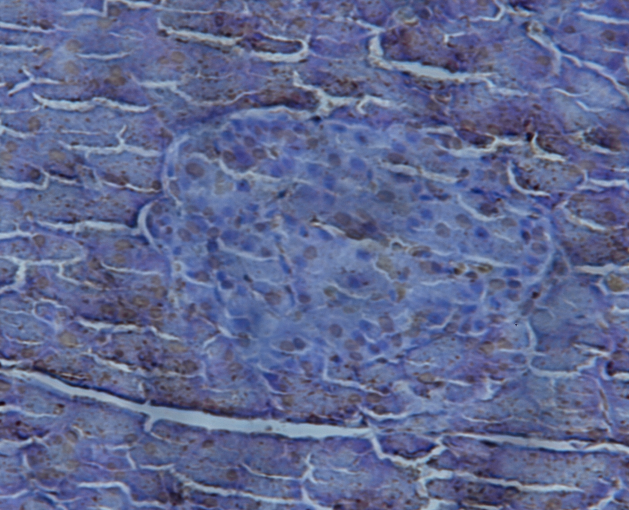

Supplement: Supplementary file 7 — Supplementary Figure 6. [file 41598_2021_98714_MOESM7_ESM.tif]

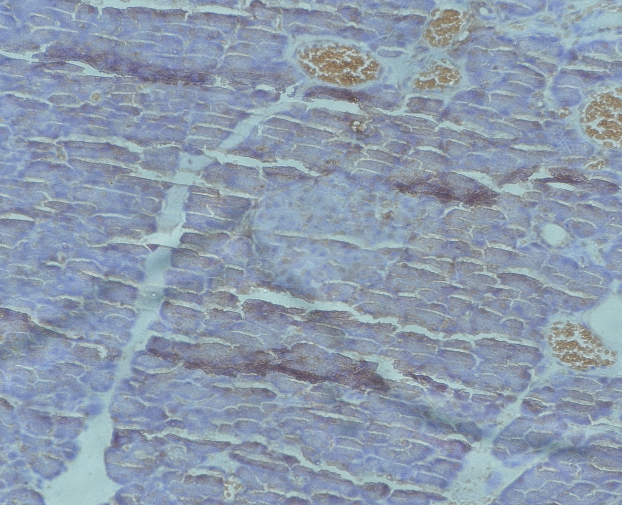

Supplement: Supplementary file 8 — Supplementary Figure 7. [file 41598_2021_98714_MOESM8_ESM.tif]

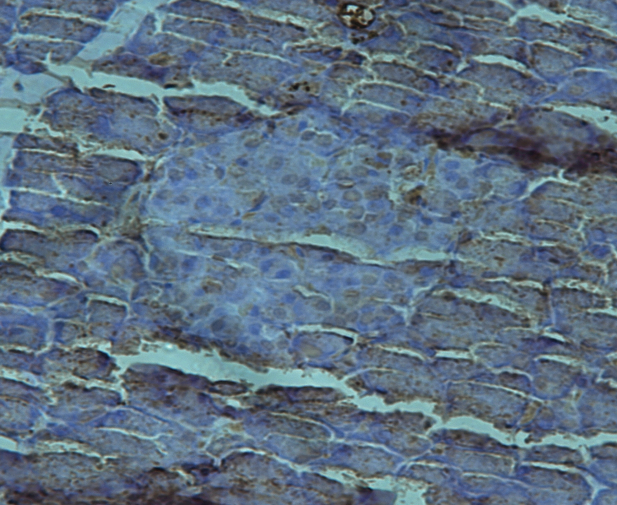

Supplement: Supplementary file 9 — Supplementary Figure 8. [file 41598_2021_98714_MOESM9_ESM.tif]

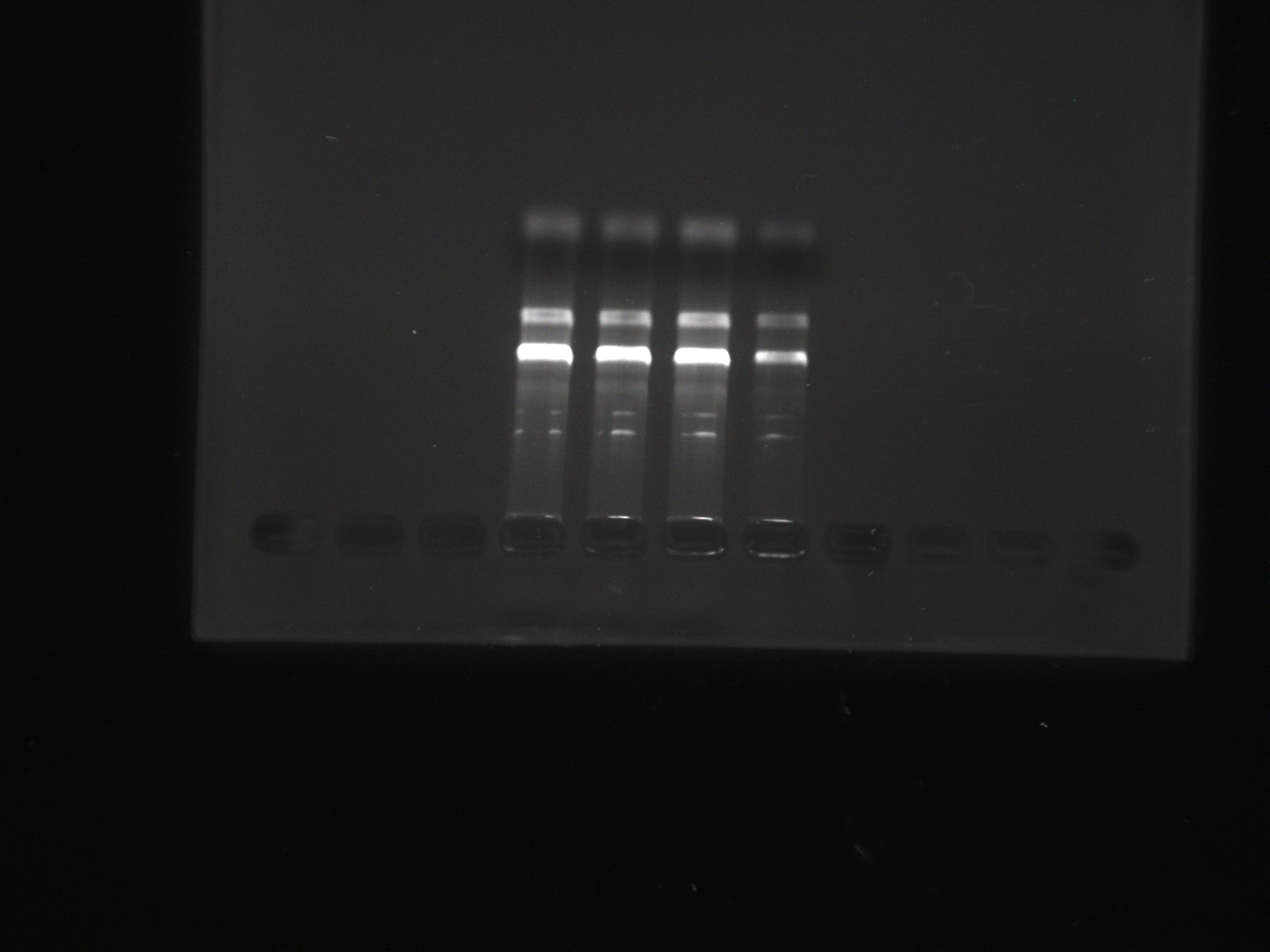

Supplement: Supplementary file 10 — Supplementary Figure 9. [file 41598_2021_98714_MOESM10_ESM.tif]

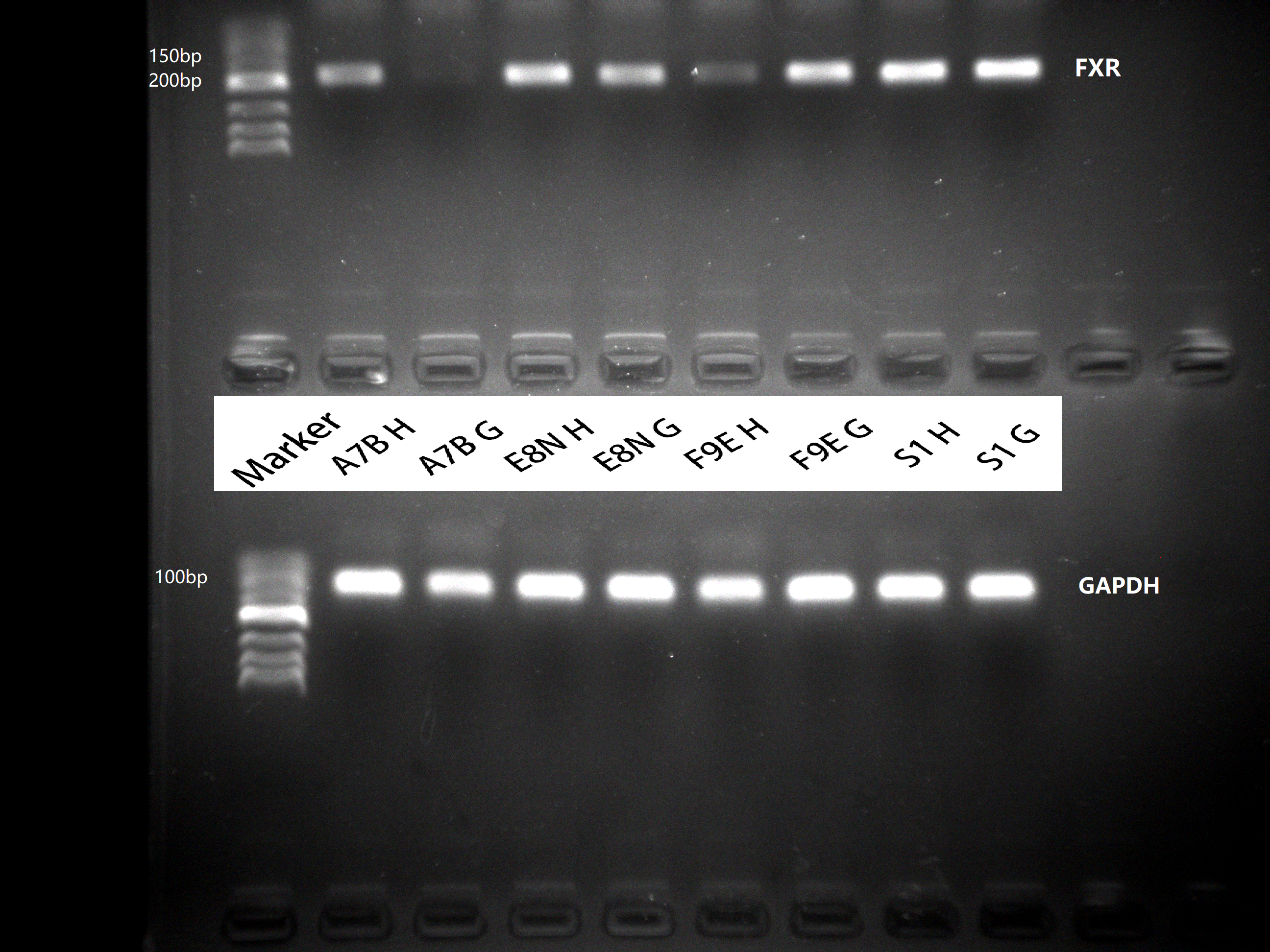

Supplement: Supplementary file 11 — Supplementary Figure 10. [file 41598_2021_98714_MOESM11_ESM.tif]

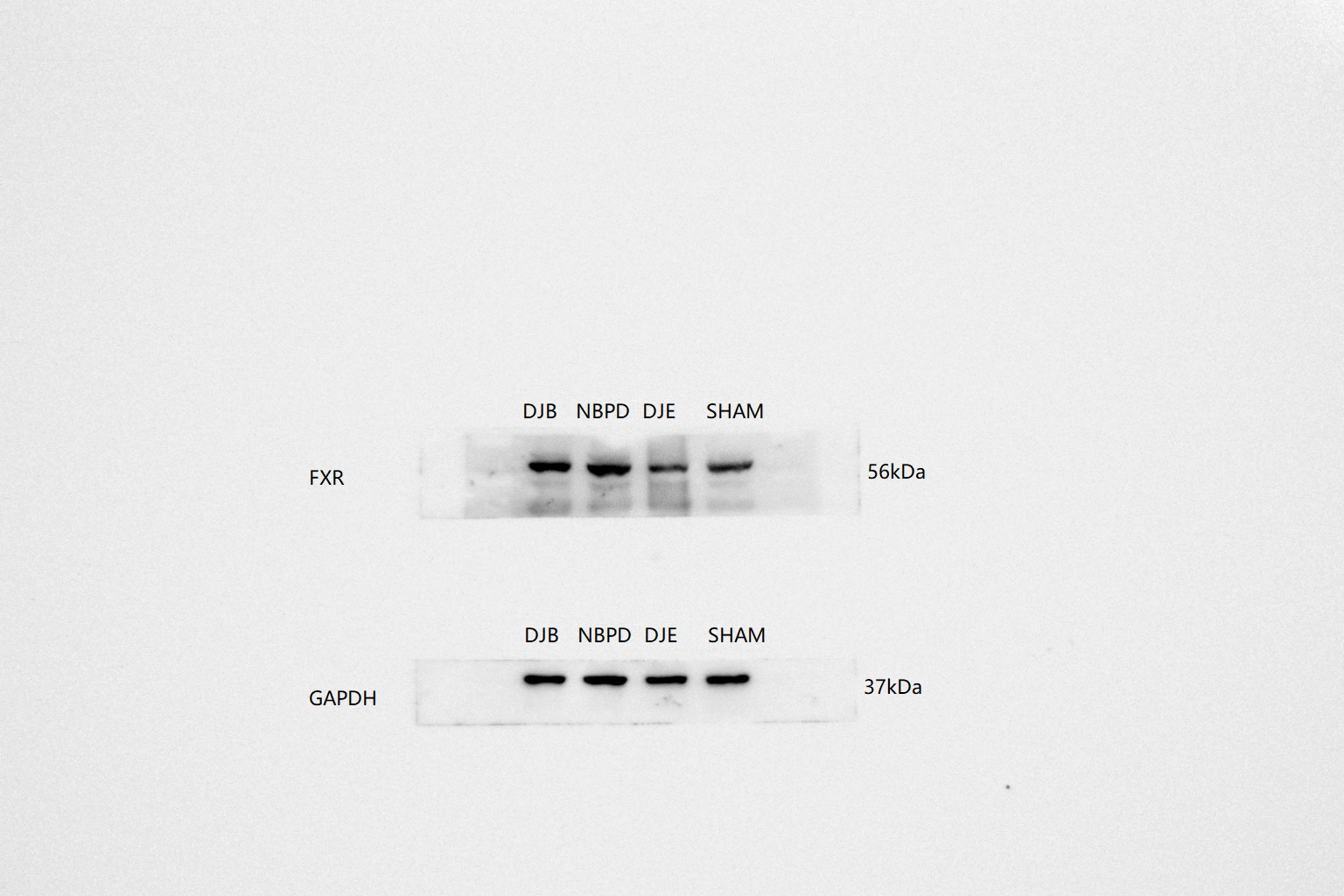

Supplement: Supplementary file 12 — Supplementary Figure 11. [file 41598_2021_98714_MOESM12_ESM.tif]
